# Mitochondria-associated membranes (MAMs) and pathologies

**DOI:** 10.1038/s41419-018-0424-1

**Published:** 2018-03-16

**Authors:** Paolo Pinton

**Affiliations:** 1 Department of Morphology, Surgery and Experimental Medicine, Section of Pathology, Oncology and Experimental Biology, Laboratory for Technologies of Advanced Therapies (LTTA), University of Ferrara, Ferrara, Italy; 2 Cecilia Hospital, GVM Care & Research, E.S: Health Science Foundation, Cotignola, Italy

Due to their typical filamentous architecture, both mitochondria and endoplasmic reticulum (ER) establish tight connections with other intracellular organelles, including nucleus and plasma-membrane, thereby modulating a wide range of cellular processes.

The main function of the ER is to control the folding of proteins and to sort them to the Golgi apparatus. However, it is also a primary sensor of intracellular stressful conditions, activating a wide number of molecular pathways that belong to a specific process named ER stress response^1^. Mitochondria are key determinant of cellular fate, by regulating both vital aspects, such as energy production and lipid biogenesis, or mediating the trigger of death programs^2^. ER and mitochondria continuously exchange messages to maintain the intracellular homeostasis. This communication is achieved by a physical association between the two organelles. Indeed, ER and mitochondria membranes touch one each other, forming a specific microdomain termed MAMs: mitochondria-associated membranes^3^. Such ER-mitochondria connection serves as a platform for the sorting of vital and dangerous signals and also operates as a structural allocation for multiple scaffold proteins and regulatory factors.

In the last years, multiple methods have been developed to dissect MAMs’ specific properties and protein composition, either using biochemical (i.e., subcellular fractionation on Percoll gradient)^4^ or fluorescent microscopy-based strategies^5, 6^. We have enormously extended our comprehension on MAMs’ dynamics, which provided new correlations between MAMs’ dysfunctions and human diseases. These pathological scenarios are characterized by alterations in the normal communication between ER and mitochondria, leading to deep metabolic defects that contribute to the progression of the disease and could be considered as real pathogenic events.

As a general introduction to MAMs in health and disease, several groups reviewed the current fundamental knowledge on the topic. Missiroli et al. first addressed the emerging role of MAMs in inflammation and in particular in the activation of the NOD-like receptor protein 3 (NLRP3) inflammasome, in the host-derived damage signals (damage-associated molecular patterns, DAMPs) generation, antiviral responses and bacterial pathogens-mediated infection^7^. Martinvalet provides a detailed overview of the vital role of the contact sites between mitochondria and ER during the development of the immune responses^8^.

Altered signaling occurring at the MAMs has been described in different cancer types. Kerkhofs et al. draw a wide-angle picture on the recent studies that connect ER-mitochondrial calcium (Ca^2+^) transfer, the role of the MAM and the proteins contained in this virtual space in influencing Ca^2+^ transfer, and how these processes contribute to cancer, and influence chemotherapy efficacy^9^.

Multiple studies revealed that mitochondria and ER play a central role in the regulation of neurological activities, and studies of MAMs properties evidenced deregulations in many neurodegenerative disorders. Area-Gomez et al. provide a detailed overview on the relationship existing between Alzheimer’s Disease (AD) and the role that mitochondria, MAMs, and lipids/cholesterol homeostasis could have in mitochondria intoxication^10^.

Gómez-Suaga et al. then discuss the main pathways, damaged in Parkinson’s disease (PD). Indeed, several PD-related proteins localize in mitochondria or MAM and have been shown to participate in ER-mitochondria signaling regulation. Likewise, PD-related mutations have been shown to damage this signaling and as ER-mitochondria associations are altered in PD^11^.

The review submitted by Lau and colleagues aims at describing the more recent data on the link between altered ER-mitochondria signaling and two neurodegenerative diseases, the fronto-temporal dementia (FTD) and the amyotrophic lateral sclerosis (ALS). Then, they focused on what may be commonly deficient in FTD and ALS^12^.

On the same topic, Bernard-Marissal et al. discuss mitochondria-ER dysfunctions and neurodegenerative diseases that affect neurons characterized by long projecting axons, i.e., ALS and hereditary motor and sensory neuropathy (HMSN) focusing on the role of Ca^2+^ homeostasis, mitochondrial dynamics, ER functioning and autophagy in neuronal survivability^13^.

Annunziata and colleagues examine the pivotal role of lipid components that influence the function and structural characteristic of the MAMs. They describe how impaired lysosomal turnover alters the lipid conformation and functional properties of the MAMs leading to neuronal cell death and neurodegeneration. In particular, they focus on the role of MAMs in the pathogenesis of GM1-gangliosidosis^14^.

The review by Delprat et al. addresses the potential role of ER-mitochondria communication in the Wolfram syndromes (WS1 and WS2). Some experimental evidence suggests a possible link between alterations clinical symptoms and alterations in Ca^2+^ homeostasis, UPR, and autophagy caused by mutations in specific proteins (WF1 and WF2, respectively) localized at the ER and MAMs^15^.

Alterations of ER-mitochondria communication have been related to metabolic diseases suggesting a pivotal role for MAMs in the control of insulin signaling and glucose utilization as discussed by Rieusset where is also analyzed the involvement of mitochondria and ER in the control of glucose homeostasis by describing the organelle-specific activities^16^.

A considerable amount of data linking MAMs to processes which might contribute to aging are presented by Janikiewicz et al. where a pivotal role of reactive oxygen species (ROS) are proposed^17^. Evaluation of the redox interplay between three organelles that produce ROS: mitochondria, ER, and peroxisomes, drawing a new ternary structure involved, called “redox triangle” is addressed by Yoboue et al. This manuscript analyzes the enzymes responsible for ROS production and also ROS degradation and signaling mediated by ROS in three compartments, ER, mitochondria and peroxisomes, assembling “redoxosomes” that sense ROS accumulations and redox imbalances at the MAMs where ROS influence the Ca2+ homeostasis acting on redox-sensitive proteins localized at the MAMs^18^.

A controversial and widely discussed topic, the effects of mitofusin-2, the outer mitochondrial membrane GTPase that, together with its close homolog mitofusin-1, mediates mitochondrial fusion and takes part in the tethering of mitochondria to other organelles, is still to be established. The review by Filadi et al. addresses this challenging issue describing the role of mitofusin-2 at the MAMs and its roles in multiple physiological processes, and disorders such as neurodegenerative disease, cardiomyopathy, obesity, diabetes, and cancer^19^.

Finally, all the reviews not only summarized the most recent studies on MAMs as hot spot signaling compartment but more importantly, they raising and also commenting on potentially controversial issues in this field of research and seemingly contradictory results.

The findings here described are only the beginning of a new role of mitochondria and ER contact sites, and more detailed analyses of the role of the MAMs in human pathologies will soon follow.
